# Supplementary material for: A Global Screen for Assembly State Changes of the Mitotic Proteome by SEC-SWATH-MS
Source: Cell Syst. 2020 Feb 26;10(2):133–155.e6. doi: 10.1016/j.cels.2020.01.001 (PMC7042714; doi:10.1016/j.cels.2020.01.001)

Q9UNN5 | FAF1_HUMAN | FAF1 UBXD12 UBXN3A CGI−03
Monomer MW [kDa]: 73.954 Monomer expected elution fraction: 43

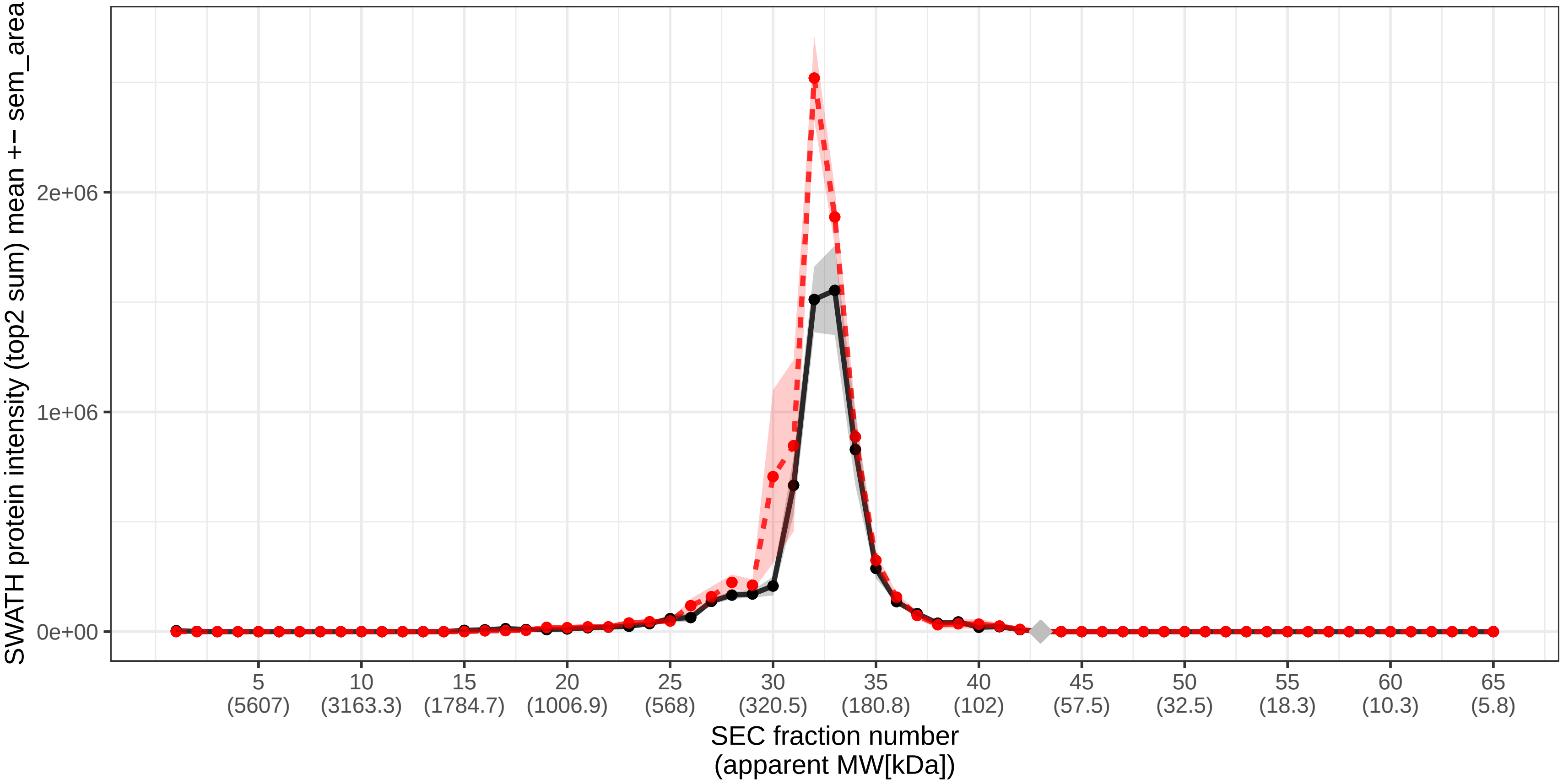

Supplement: Data S1. SEC-SWATH-MS Protein Chromatograms, Related to Figure 1 [file mmc6.zip › SECchrom_Q9UNN5_FAF1_HUMAN_FAF1_UBXD12_UBXN3A_CGI-03.pdf]
